# A Federated Hierarchical DQN-Based Distributed Intelligent Anti-Jamming Method for UAVs

**DOI:** 10.3390/s26010181

**Published:** 2025-12-26

**Authors:** Dadong Ni, Shuo Ma, Junyi Du, Yuansheng Wu, Chengxu Zhou, Haitao Xiao

**Affiliations:** 1 National Key Laboratory of Complex Aviation System Simulation, Chengdu 610036, China; 2 The Key Laboratory of Intelligent Network and Network Security (Ministry of Education), College of Information and Communication Engineering, Xi’an Jiaotong University, Xi’an 710049, China

**Keywords:** anti-jamming intelligent decision-making, deep federated learning, hierarchical DQN, distributed decision-making

## Abstract

In recent years, with the rapid development of intelligent communication technologies, anti-jamming techniques based on deep learning have been widely adopted in unmanned aerial vehicle (UAV) systems, yielding significant improvements. Most existing studies primarily focus on intelligent anti-jamming decision-making for single UAVs. However, in UAV swarm systems, single-agent decision models often suffer from data isolation and inconsistent frequency usage decisions among nodes within the same task subnet, caused by asynchronous model updates. Although data sharing among UAVs can partially alleviate model update issues, it introduces significant communication overhead and data security challenges. To address these problems, this paper proposes a novel multi-UAV cooperative intelligent anti-jamming decision-making method, termed Federated Learning-Hierarchical Deep Q-Network (FL-HDQN). First, an adaptive model synchronization mechanism is integrated into the federated learning framework. By sharing only local model parameters instead of raw data, UAVs collaboratively train a global model for each task subnet. This approach ensures decision consistency while preserving data privacy and reducing communication costs. Second, to overcome the curse of dimensionality caused by multi-domain interference parameters, a hierarchical deep reinforcement learning model is designed. The model decouples multi-domain optimization into two levels: the first layer performs time–frequency domain decisions, and the second layer conducts power and modulation-coding domain decisions, ensuring both real-time performance and decision effectiveness. Finally, simulation results demonstrate that, compared with state-of-the-art intelligent anti-jamming models, the proposed method achieves 1% higher decision accuracy, validating its superiority and effectiveness.

## 1. Introduction

With the rapid development of unmanned aerial vehicle (UAV) technology and intelligent communication systems, UAVs have been widely deployed in a variety of applications, including military reconnaissance, emergency rescue, logistics transportation, environmental monitoring, and communication relay. In particular, UAV swarm systems, owing to their high degree of cooperation, flexibility, and task redundancy, are gradually becoming an essential component of future airborne information systems and intelligent operational frameworks. In this context, the reliability and security of communications in UAV swarms have attracted extensive attention from both the academic and engineering communities. However, due to the open nature of wireless communication environments and the high mobility of UAV nodes, UAV swarm communications are highly vulnerable to various malicious jamming and spoofing attacks, such as broadband jamming, co-channel interference, and intelligent jamming and deceptive attacks based on cognitive radio techniques. These attacks not only significantly degrade communication link quality, but may also lead to communication outages within task-oriented subnetworks, thereby posing serious threats to the mission execution capability and overall system security of UAV swarm networks. To address the highly dynamic characteristics and frequently changing network topologies in UAV swarm operations, adopting a distributed network architecture can effectively enhance the survivability and robustness of UAV swarm networks. Moreover, achieving secure and reliable information transmission is critical for mission completion, making reliable inter-UAV communication a key research focus.

In recent years, the emergence of intelligent and cognitive jamming has posed significant challenges to UAV anti-jamming communications. Effectively countering such intelligent jamming has become a major research focus. With the rapid development of artificial intelligence algorithms, intelligent decision-making methods have been increasingly employed in anti-jamming communications. These methods typically use reinforcement learning or game-theoretic approaches to adaptively select optimal channels and transmission power from the frequency or power domain to resist jamming [1]. From the frequency-domain perspective, reference [2] modeled the channel selection problem as a multi-armed bandit (MAB) model, where the channel corresponding to the optimal arm is selected to avoid interference. Building upon MAB theory, reference [3] proposed a channel selection algorithm based on the upper confidence bound (UCB), which effectively reduces collision probability and regret in dynamic spectrum access. Zeng et al. [4] proposed a reinforcement learning method with prior knowledge to address spectrum access for cognitive users under rapidly varying interference environments. Their approach employed a reliability quantification mechanism to guide the use of prior knowledge during learning. In the power domain, reference [5] applied collaborative Q-learning (QL) for channel selection, which improved secure data transmission capacity but suffered from slow convergence. Reference [6] enhanced the efficiency of channel selection through a competitive DQN algorithm with prioritized experience replay in a centralized training environment. For large-scale channel scenarios, references [7,8] adopted an actor-critic (AC) algorithm to select secure channels. However, due to real-time parameter updates, the strong dependency between the actor and critic networks reduced algorithm stability. From the power-domain perspective, references [9,10,11] utilized game theory to model cognitive anti-jamming networks under low interference power conditions. By analyzing the competitive relationship between players, the Nash equilibrium was derived to obtain the optimal transmission power for each user. To further enhance the capability of countering intelligent jamming, multi-domain joint anti-jamming approaches have been proposed [12]. Reference [13] considered both the power and frequency domains. It first adopted a Stackelberg game to assess interference levels in the power domain and then applied an MAB algorithm for frequency-domain channel selection. Similarly, reference [14] categorized interference levels into mild, moderate, and severe. For moderate interference, a game-theoretic strategy in the power domain was adopted, whereas for mild interference, a frequency-domain algorithm optimized using the AC method was employed to avoid jamming. Zhang et al. [15] designed a dual-mode selection strategy integrating multi-user communication and deception modes. By dynamically adjusting channel and power selections, their method effectively countered both conventional and perception-tracking jamming. Yao et al. [16] investigated anti-jamming defense in multi-user scenarios and proposed a collaborative multi-agent anti-jamming algorithm (CMAA). Their method modeled the defense problem within a Markov game framework, enabling cooperative decision-making among users to resist jamming more effectively. Zhu et al. [17] addressed the spectrum anti-jamming problem for uplink transmission devices by learning frequency-domain channel allocation strategies without prior knowledge of the temporal-frequency distribution of interference. They adopted a multi-agent reinforcement learning framework using value function approximation to sequentially optimize the configuration policies of IoT devices. Zhou et al. [18] proposed a fast anti-jamming scheme for distributed wireless networks based on intra-domain knowledge reuse. By introducing a dual-simulation relationship to measure the similarity between different state-action pairs in the anti-jamming problem, they built a bridge for knowledge transfer among nodes, enabling each node to learn the characteristics of dynamic environments independently. Qi et al. [19] designed a cooperative anti-jamming strategy in which a friendly jammer intentionally sacrifices part of the ally’s performance to disrupt the opponent’s communication. The interaction between the friendly jammer and other nodes was formulated as a Stackelberg game, effectively suppressing adversarial communications while minimizing collateral impact on other nodes. Elleuch et al. [20] proposed a distributed learning algorithm to mitigate interference in multi-user networks. Their approach relied on secure communication links for information exchange among users and introduced a cross-check Q-learning algorithm for interference mitigation. Peng et al. [21] developed an improved reinforcement learning algorithm based on multi-parameter optimization to handle multiple interference sources between UAV swarms and base stations. Huang et al. [22] investigated joint relay and channel selection in multi-relay anti-jamming communication systems. Without prior knowledge of interference patterns or relay node distributions, they formulated the relay and channel selection problem as a Markov decision process (MDP) and proposed a reinforcement learning algorithm for joint relay and channel selection to enhance system robustness against interference. Reference [23] proposed a hierarchical deep reinforcement learning algorithm to address the frequency selection problem in broadband jamming environments. The method divides the selection into two steps—first choosing the frequency band and then selecting the specific frequency within that band—effectively avoiding multiple jamming attacks and achieving satisfactory throughput with reduced computational complexity. Reference [24] investigated spatial anti-jamming communication in heterogeneous Internet of Satellites and proposed a Stackelberg game–based scheme combined with deep reinforcement learning to achieve low-cost and effective anti-jamming routing under dynamic jamming environments. Reference [25] proposed a deep Q-learning–based frequency hopping strategy to combat probabilistic multi-channel jamming in wideband communications without requiring prior knowledge of jamming patterns. Reference [26] proposed a multi-agent deep reinforcement learning–based UAV swarm communication scheme to jointly optimize relay selection and power allocation against jamming under unknown network and channel conditions. Reference [27] modeled the multi-user anti-jamming problem in wireless sensor networks as a stochastic game and proposed a joint multi-agent reinforcement learning–based anti-jamming algorithm to achieve effective channel selection under intelligent multi-channel blocking jamming.

However, the aforementioned studies mainly focus on single-UAV or UAV-pair scenarios, without considering the practical situation in which multiple UAVs usually operate cooperatively in formations to perform complex missions. In UAV swarm applications, existing intelligent anti-jamming decision-making methods face several critical challenges:

1. Decision inconsistency: during mission execution, UAVs operate under diverse conditions such as varying positions, flight altitudes, and environmental characteristics. As a result, the perceived electromagnetic environment data exhibit significant heterogeneity, leading to inconsistent frequency-use decisions among UAVs. This inconsistency degrades the overall coordination efficiency and operational effectiveness of the swarm.
2. Data isolation: when each UAV trains its decision model solely on locally perceived data, the limited data volume may cause insufficient model training and updating. Moreover, the electromagnetic environment is highly dynamic and complex; data collected by a single UAV are often too limited to capture the full range of environmental variations. Consequently, the trained model may quickly lose effectiveness when encountering new interference types, resulting in poor adaptability to rapidly changing environments.
3. Data security risks: sharing local data among UAVs can mitigate the data isolation problem and improve model training. However, such data sharing compromises user privacy and poses serious risks of data interception by adversaries. It also introduces additional communication overhead, making it difficult to maintain both efficiency and data confidentiality.
4. Curse of dimensionality in multi-domain joint decision-making: most existing multi-domain anti-jamming decision models perform joint optimization in two domains (typically frequency and power or time and frequency) to enhance anti-jamming capability. However, they often overlook the joint optimization of modulation-coding parameters, which is crucial for maximizing transmission rate while maintaining robustness against interference. Moreover, multi-domain integration dramatically expands the state-action space, leading to exponential growth in learning complexity, slower convergence, and reduced decision-making efficiency.

To address the limitations identified in existing studies, this paper proposes a distributed multi-UAV cooperative intelligent anti-jamming framework termed FL-HDQN. Unlike conventional centralized or single-agent learning approaches, the proposed framework explicitly considers decision consistency and data privacy in distributed UAV swarms by integrating federated learning with deep reinforcement learning. In contrast to existing federated learning–based schemes that typically employ single-layer policies, FL-HDQN incorporates a hierarchical DQN structure to cope with the multi-domain decision-making complexity in anti-jamming scenarios. Specifically, by sharing only local model parameters rather than raw observations, UAVs collaboratively train global decision models while preserving data privacy and reducing communication overhead. An adaptive model synchronization mechanism is further introduced to mitigate the impact of heterogeneous computation capabilities and time-varying network conditions, which are often overlooked in existing federated or multi-agent reinforcement learning frameworks. Moreover, the hierarchical learning structure decomposes the multi-domain anti-jamming problem into time–frequency decision-making and power–modulation-coding optimization, effectively alleviating the curse of dimensionality and improving learning stability. These characteristics distinguish the proposed FL-HDQN from recent federated or hierarchical reinforcement learning approaches for UAV anti-jamming communications.

The main contributions of this work are summarized as follows:

1. Federated multi-UAV decision-making: this study introduces the federated learning paradigm into DQN for multi-UAV cooperative anti-jamming scenarios. UAVs share only local model parameters without exchanging raw data to collaboratively train a global anti-jamming decision model. Each UAV then performs local decision-making using the jointly trained model, achieving consistent distributed decisions across the swarm while mitigating the generalization issues caused by isolated local data.
2. Adaptive model synchronization mechanism: to address differences in local training times and communication delays caused by heterogeneous computation capabilities and network conditions, an adaptive model synchronization strategy is proposed to facilitate effective federated aggregation.
3. Hierarchical multi-domain reinforcement learning: to overcome the curse of dimensionality in multi-domain optimization, a hierarchical reinforcement learning framework is designed. The multi-domain problem is decoupled into multiple sub-tasks: the time–frequency domain is treated as one sub-task, while power and modulation-coding form another. Each sub-task corresponds to a separate policy layer, and the layers cooperate to achieve the overall anti-jamming objective.
4. Experimental validation: the proposed method is evaluated under multiple interference scenarios. Simulation results demonstrate the superiority and effectiveness of the FL-HDQN approach.

## 2. Model and Formulation

### 2.1. System Model

**Figure 1:**
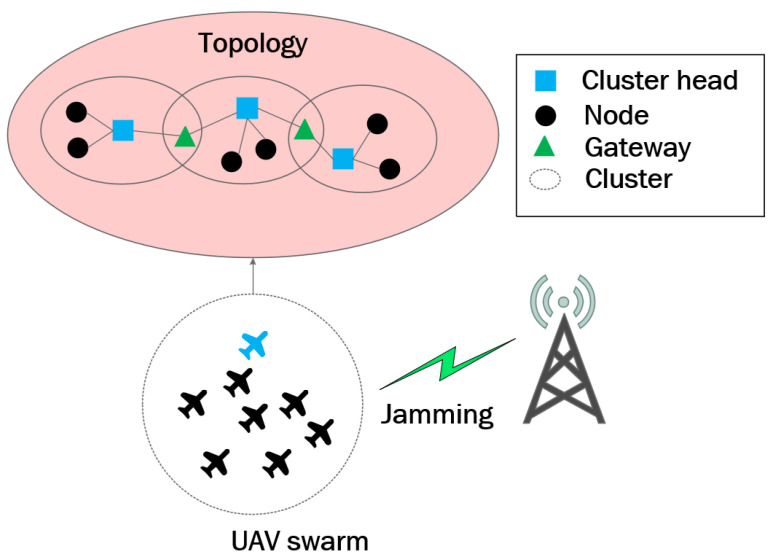
Overall model of unmanned aircraft fleet.

This study adopts a clustered UAV swarm architecture. When the number of UAVs exceeds eight, a hierarchical multi-cluster mobile ad hoc network (MANET) is used to organize the swarm. In this hierarchical network, UAVs are grouped into different clusters, and each cluster is a task subnet. The proposed FL-HDQN framework is designed to operate within a single task subnet, where a group of UAVs collaboratively execute the same communication task and participate in federated learning under a shared coordination mechanism. Within each cluster, time-division multiple access (TDMA) is employed, while frequency-division multiple access (FDMA) is used for inter-cluster communication. Each cluster is coordinated by a cluster head, which manages intra-cluster information and forwards critical data and commands to other cluster heads or the backend control system. This hierarchical structure not only reduces communication complexity but also allows the UAV swarm to respond more flexibly to environmental changes and mission requirements, significantly enhancing the efficiency and reliability of coordinated operations. The overall UAV swarm model is illustrated in Figure 1. In this study, the collaborative anti-jamming decision-making process is conducted at the cluster level, ensuring that all UAVs within a cluster make consistent frequency-use decisions. This approach effectively prevents inconsistent channel allocation policies caused by inter-UAV data heterogeneity or data isolation.

**Figure 2:**
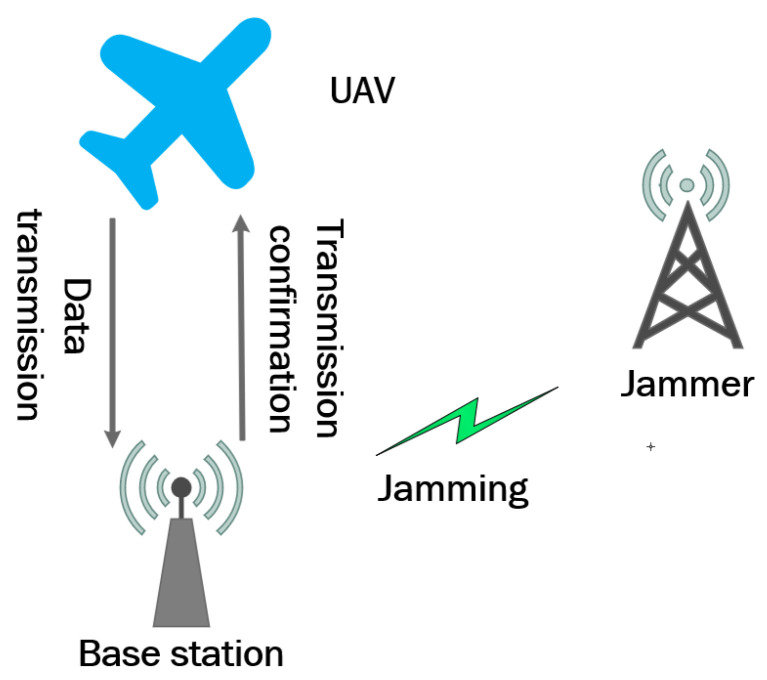
Anti-jamming model for a single UAV in a cluster.

The individual anti-jamming model for UAVs within a cluster is illustrated in Figure 2. Each UAV acts as the source of a communication link, transmitting data to the receiver during its assigned communication time slot. The jammer aims to disrupt the communication between the UAV and the receiver by transmitting interference signals, which can block or distort the UAV’s transmission. Such interference may result in transmission failures or prevent the receiver from correctly decoding the transmitted information.

### 2.2. Interference Model

During mission execution in complex environments, UAVs often encounter various types of electronic interference due to the rapid development of electronic technologies [28]. This section introduces several typical communication jamming signals, including single-tone jamming, multi-tone jamming, tracking jamming and sweeping jamming.

Single-tone jamming refers to interference in a communication system caused by a signal at a single frequency that affects data transmission. Such interference typically originates from specific electronic devices or system leakage and produces a continuous, fixed-frequency disturbance in the received signal, thereby degrading communication quality. The mathematical expression for single-tone jamming is given in  (1).

(1) $$J\left(t\right)=Aexp\left(j(2\pi f_{0}t+\varphi )\right)$$

where *A* denotes the amplitude of the single-tone jamming, $f_{0}$ denotes the frequency of the interference signal, and φ denotes the initial phase.

Multi-tone jamming refers to the scenario in which the jammer generates multiple interference signals at different frequencies within a specific band, effectively simulating a combination of single-tone jamming signals. This approach allows the power and frequency of each interference signal to be adjusted according to operational requirements, thereby selectively affecting the received signals of the communication system. Multi-tone jamming poses additional challenges to communication systems, as it introduces interference across multiple frequencies, complicating both interference suppression and signal recovery. The mathematical expression for multi-tone jamming is given in (2).

(2) $$J\left(t\right)=\sum_{m=1}^{M}A_{m}\exp\left(j(2\pi f_{m}t+\varphi_{m})\right)$$

where $A_{m}$ denotes the amplitude of the *m*-th tone, $f_{m}$ denotes the frequency of the *m*-th tone, and $\varphi_{m}$ is the initial phase of the *m*-th tone.

Sweeping jamming involves rapidly varying the frequency of the interference signal to cover a wide frequency band, aiming to disrupt or degrade the received signals of a specific communication system. By continuously scanning multiple frequency points within a short period, this type of jamming prevents the receiver from reliably locking onto the intended signal, thereby reducing communication efficiency or causing communication interruptions. Sweeping jamming is particularly challenging for protected communication systems, as it requires the receiver to possess high adaptability and strong interference recognition capabilities. The mathematical expression for sweeping jamming is given in (3).

(3) $$J\left(t\right)=\sum_{m=1}^{M}A_{m}\exp\left(j(2\pi f_{0}t+\pi kt^{2}+\varphi_{m})\right)$$

where *A* denotes the amplitude of the linear sweeping jamming, $f_{0}$ denotes the initial frequency of the sweep, and φ denotes the initial phase.

Tracking jamming targets a specific communication or radar signal by continuously monitoring the target’s instantaneous frequency and synchronously adjusting the jammer’s frequency so that the interference remains aligned with the target signal. By maintaining frequency synchronization with the target, tracking jamming can effectively disrupt or block the target system’s normal operation. This form of jamming is especially destructive for systems that depend on precise signal reception.

To effectively jam frequency-hopping (FH) communication systems, a tracking jammer must not only deliver sufficiently high power but also satisfy a set of operational requirements. These requirements, summarized in (4), include the ability to rapidly detect and identify the target’s hop frequency and to quickly adjust the jammer’s own emission frequency so that its hops remain temporally and spectrally aligned with the target’s frequency hops. Meeting these conditions ensures the jammer can follow the target’s frequency jumps and maintain disruptive overlap with the intended signal.

(4) $$\left(d_{1}+d_{2}\right)\leq \eta \left(\left(T_{h}-T_{q}\right)-T_{p}\right)\nu -d_{0}$$

where ν denotes the signal propagation speed, $d_{1}$ denotes the distance from the jammer to the transmitter, $d_{2}$ denotes the distance from the jammer to the receiver, and $d_{0}$ denotes the distance between the transmitter and receiver of the target signal. $T_{p}$ is the analysis time required by the jammer after intercepting the target signal, $T_{q}$ denotes the frequency synthesizer’s switching time, $T_{h}$ is the hop period, and η denotes the system’s anti-jamming tolerance factor.

## 3. Methods

**Figure 3:**
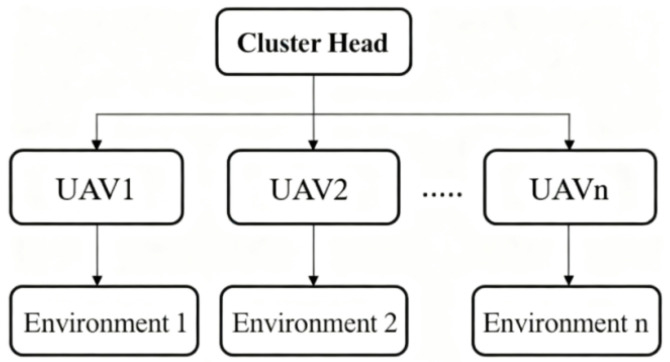
Federated Learning Framework.

In this section, a federated learning-based multi-domain joint intelligent anti-jamming decision-making method for UAV swarms is proposed to address the communication challenges arising during cooperative task execution. The overall framework of the federated learning model is illustrated in Figure 3. Each UAV first performs self-training within its local environment. Instead of sharing raw data, selected UAVs transmit only their trained model parameters to the cluster head. The cluster head then conducts model aggregation and task distribution through an Adaptive Model Synchronization Mechanism (AMSM), thereby achieving decision consistency while ensuring data security. During the self-training process, a hierarchical deep reinforcement learning (DRL) approach is employed, in which the first layer handles time-frequency domain decisions, and the second layer manages power and modulation-coding domain decisions. This hierarchical structure effectively mitigates the curse of dimensionality caused by complex action spaces and enhances the training efficiency.

### 3.1. Adaptive Model Synchronization Mechanism

In real-world scenarios, UAV swarms are typically deployed in complex and dynamically changing communication environments. Under such conditions, significant disparities often exist in the network states experienced by different UAVs, and temporary offline situations are not uncommon in practical operations. Moreover, UAVs may frequently lose connection due to factors such as battery depletion or signal interference. In addition, the onboard computing hardware of each UAV varies, resulting in differences in data processing and learning speeds. These factors collectively lead to variations in the time required for individual UAVs to complete local model training. Therefore, a flexible training and communication mechanism is essential to enable UAVs to continue local learning even when communication with the cluster head or other UAVs is interrupted.

**Figure 4:**
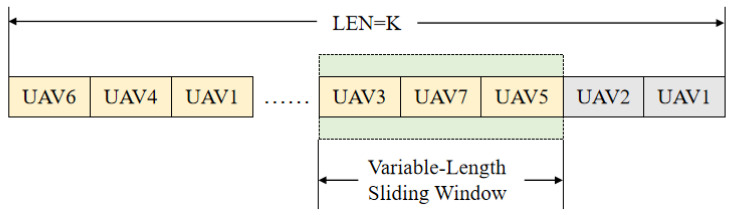
Schematic diagram of the model buffer.

In this study, model selection and utilization are performed based on the adaptive model synchronization mechanism (AMSM). Within this framework, each UAV independently conducts local training and uploads its updated model parameters to the cluster head upon completion. Instead of waiting for all UAVs to finish uploading, the cluster head triggers model aggregation once a sufficient number of local model updates has been received, thereby improving aggregation efficiency and better accommodating the dynamic characteristics of practical deployment environments. Specifically, the cluster head maintains a model buffer, denoted as Buffer, to temporarily store the locally updated model parameters collected in each training round. To adaptively determine when to perform aggregation, a variable-length sliding window is introduced to estimate the expected number of model updates per round. The cluster head records the actual number of received local models in each round and stores these values in a historical record buffer. If the number of received models in the current round matches the expected value, the expected number remains unchanged. Otherwise, the expected number is updated as the average of the most recent x recorded values. This sliding-window-based adjustment enables the synchronization process to adapt to variations in UAV participation caused by mobility, channel conditions, or intermittent interference. The architecture of the model buffer and the sliding window mechanism is illustrated in Figure 4.

Furthermore, two key timing parameters are defined at the cluster head: the maximum waiting time per federated round, denoted as $wait_{\max}$, and an additional waiting duration $wait_{\mathrm{rec}}$ that is activated after the expected number of local models has been collected.

At the beginning of each federated learning round, the cluster head broadcasts the current global model to all online UAVs for local training. After completing local updates, UAVs upload their model parameters to the cluster head, which continuously monitors the status of the model collection buffer.

Once the number of received local models reaches a predefined expected threshold, the cluster head initiates an additional waiting period of duration $wait_{\mathrm{rec}}$. If no further model updates are received during this interval, the aggregation process is immediately triggered to generate the global model for the next round.

Alternatively, if the expected number of models is not reached within the maximum waiting time $wait_{\max}$, the cluster head proceeds with model aggregation to ensure learning continuity.

Accordingly, the aggregation trigger at round *t* is formally defined as

(5) $$T_{t}=\left\{\begin{array}{cc} 1, & \mathrm{if}\;\left(N_{t}\geq {\hat{N}}_{t}\;\wedge \;\Delta t\geq wait_{\mathrm{rec}}\right),\\ 1, & \mathrm{if}\;\Delta t\geq wait_{\max},\\ 0, & \mathrm{otherwise}, \end{array}\right.$$

where $N_{t}$ denotes the number of local model updates received by the cluster head in round *t*, ${\hat{N}}_{t}$ represents the expected number of models for aggregation, and Δt is the elapsed waiting time since the beginning of the current round. $T_{t}=1$ indicates that the aggregation process is triggered, while $T_{t}=0$ means that the cluster head continues waiting for additional updates.

This design effectively mitigates update delays caused by heterogeneous computational capabilities and intermittent connectivity among UAVs, preventing slow or disconnected nodes from dominating the overall learning process.

If the maximum waiting time expires and the buffer remains empty, the cluster head defaults to using the previous global model as the latest version, thereby preventing training stagnation. This adaptive synchronization strategy ensures the continuity and stability of training while significantly reducing idle waiting time and enhancing the overall learning efficiency of the swarm.

**Figure 5:**
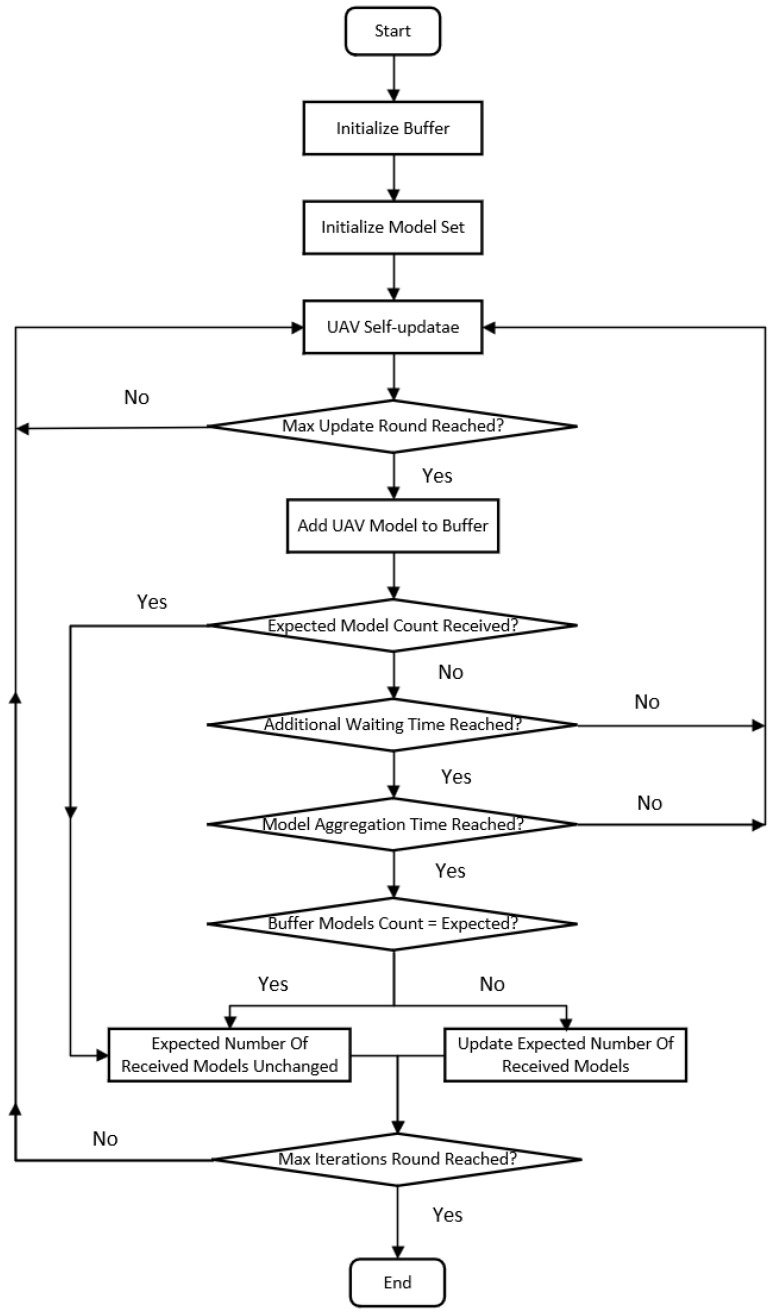
Flowchart of Adaptive Model Synchronization Mechanism.

The flowchart of the adaptive model synchronization mechanism is illustrated in Figure 5. Initially, the cluster head initializes a buffer for storing UAV model parameters and sets an initial expected model count. UAVs then perform self-updates, and once the maximum update iteration is reached, the model parameters are uploaded to the cluster head. The cluster head continuously receives model parameters from UAVs and adaptively adjusts the expected number of received models based on both the actual number of received updates and the processing time, until the maximum number of training iterations is completed.

### 3.2. Cluster-Head-Based Task Allocation Model

**Figure 6:**
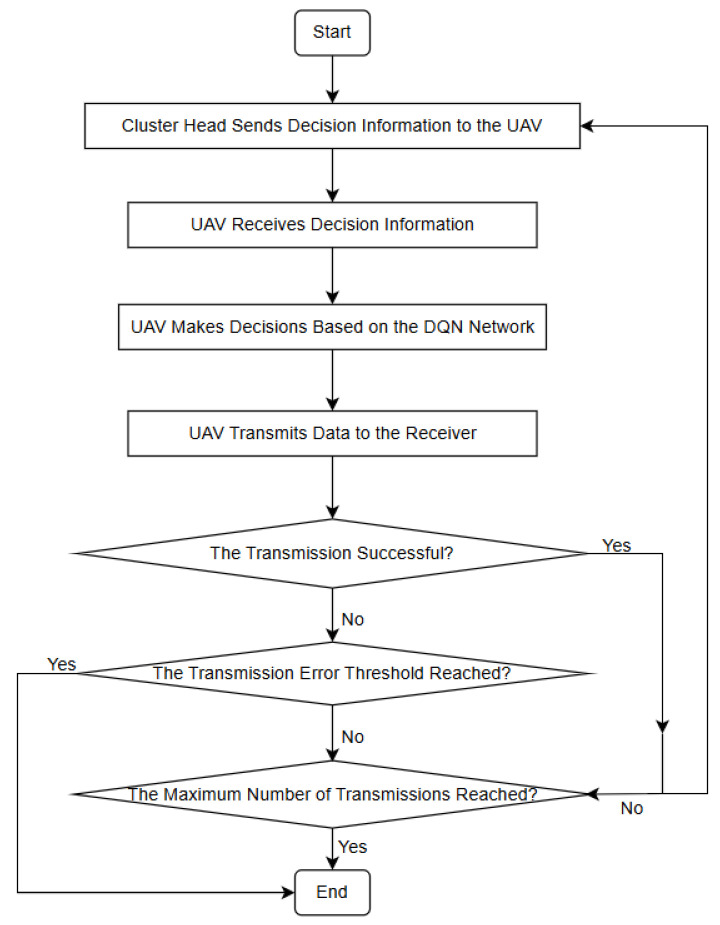
Cluster Head Task Distribution Decision Flowchart.

In addition to model updating and distribution, the cluster head is also responsible for disseminating task information to each UAV. The decision-making process for cluster-head task allocation is illustrated in Figure 4, Figure 5 and Figure 6. These task messages specify which UAV is responsible for transmitting information and which UAV is designated to receive it, thereby ensuring clear task assignment and coordinated communication execution within the swarm. After distributing the updated model parameters to the UAVs within the cluster, the cluster head proceeds to assign corresponding communication tasks to individual UAVs. During the communication process, each UAV performs its assigned task within a predefined maximum number of communication attempts. If the number of transmission errors remains below the specified threshold, denoted as error_count, the UAV continues executing the current communication task until the maximum number of communication attempts is reached. However, once the number of transmission errors exceeds this threshold, it indicates that the current communication strategy may no longer be effective. In such cases, the cluster head immediately terminates the ongoing task process and instructs the UAVs to resume model training for communication optimization.

This mechanism not only enhances the overall communication efficiency and anti-jamming capability of the UAV swarm but also reduces the risk of data leakage through centralized decision management. By maintaining consistency in both model parameters and communication strategies across the network, the proposed framework ensures that UAVs can rapidly and efficiently accomplish their missions even under highly dynamic and interference-prone environments. The federated learning-based cluster communication management method thus provides a novel and practical solution for UAV swarm operations in complex real-world scenarios.

The overall flow of the hierarchical reinforcement learning algorithm is depicted in Figure 6.

### 3.3. Hierarchical DQN-Driven Local Decision Model for UAVs

**Figure 7:**
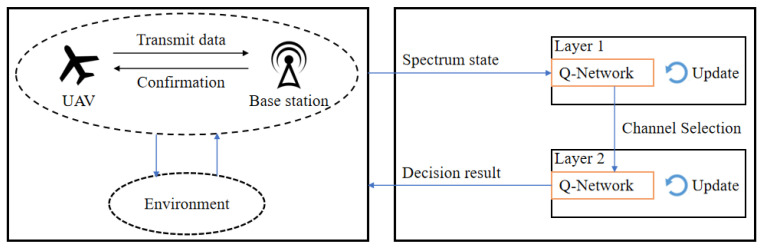
Framework of the anti-jamming method based on hierarchical reinforcement learning.

This study considers the frequency domain, power domain, and coding domain for optimizing UAV swarm communications. Although this approach reduces energy consumption and enhances transmission rates, the enlarged action space significantly slows the convergence speed of the algorithm. To accelerate convergence and improve anti-jamming performance, this paper proposes an anti-jamming model based on a hierarchical reinforcement learning (HRL) framework. The proposed hierarchical design is motivated by both communication and learning considerations. From a communication standpoint, anti-jamming decisions naturally follow a sequential structure, where frequency selection prioritizes interference avoidance, followed by power and modulation adaptation for interference mitigation. From a learning standpoint, decomposing the joint decision process into hierarchical sub-policies effectively reduces the action space dimensionality, alleviates learning complexity, and improves convergence stability compared with flat joint optimization. The proposed model decomposes the reinforcement learning process into two primary hierarchical levels: the first level corresponds to the frequency-domain layer, and the second level corresponds to the power-domain and coding-domain layer. The overall process of the proposed HRL-based anti-jamming framework is illustrated in Figure 7. In the model design, the DQN process is divided into two layers. The first layer focuses on channel selection, where the primary task is to identify and select the optimal communication channel with minimal noise and interference from the set of available channels, thereby prioritizing interference avoidance at the frequency domain. When interference cannot be completely avoided through channel selection alone, the decision output of the frequency-domain layer is passed to the second layer as a critical state input, where power control and modulation–coding decisions are further optimized to enhance communication robustness under residual interference. Based on this contextual information, the agent adjusts transmission power and selects the appropriate modulation and coding scheme. This hierarchical design ensures that communication strategies are determined under the most suitable environmental conditions, thereby significantly improving communication efficiency and reliability.

The MDP model of the first layer in the proposed hierarchical reinforcement learning framework is defined as follows:

(1) **State Space:** The current state represents the spectrum condition, which is sensed through energy detection. Each channel’s state is initialized to 0; a state value of 1 indicates that the channel is occupied by noise or interference, and 0 otherwise. If there are *c* channels in total, the size of the state space is $2^{c}$.

(2) **Action Space:** The action corresponds to the selection of available channels, denoted as C={1,2,3,…,c}, where *c* is the total number of available channels. Thus, the size of the action space is *c*.

(3) **State Transition Probability:** The transition probability is defined as P:S×A×S→[0,1].

(4) **Discount Factor:** 0<γ≤1.

(5) **Reward Function:** The reward function for the first layer is defined as in Equation (6):

(6) $$R=\left\{\begin{array}{cc} 1, & \mathrm{successful}\;\mathrm{transmission}\\ 0, & \mathrm{unsuccessful}\;\mathrm{transmission} \end{array}\right.$$

In Equation (6), after data transmission at time *t*, the bit error rate (BER) is calculated. If the BER is less than $10^{-5}$, the transmission is considered successful and the reward value is set to 1; otherwise, the reward is 0.

The MDP model of the second layer in the proposed hierarchical reinforcement learning framework is defined as follows:

(1) **State Space:** The state corresponds to the communication channel selected in the first layer. All channels are initialized to 0, and the selected channel is set to 1. If there are *c* channels in total, the size of the state space is *c*.

(2) **Action Space:** The action includes transmission power and modulation scheme selection. The power level set is P={1,2,…,p}, where *p* is the maximum power level, and the modulation set is M={1,2,…,m}, where *m* is the maximum modulation order. Therefore, the total size of the action space is p×m.

(3) **State Transition Probability:** The transition probability is defined as P:S×A×S→[0,1].

(4) **Discount Factor:** 0<γ≤1.

(5) **Reward Function:** The reward function for the second layer is defined as in (7):

(7) $$R=\left\{\begin{array}{cc} \alpha \times \mathrm{power}+\beta \times \mathrm{modulation}, & \mathrm{successful}\;\mathrm{transmission}\\ 0, & \mathrm{unsuccessful}\;\mathrm{transmission} \end{array}\right.$$

In Equation (7), power denotes the transmission power level, and modulation represents the modulation order level. The coefficients α and β are the weighting factors corresponding to the transmission power level and the modulation order level, respectively, with α>β. The objective is to maximize the data transmission rate by sequentially adjusting the transmission power and the modulation level. If the BER is greater than $10^{-5}$, the transmission is considered successful, and the reward is defined as α×power+β×modulation; otherwise, the reward is set to 0.

**Figure 8:**
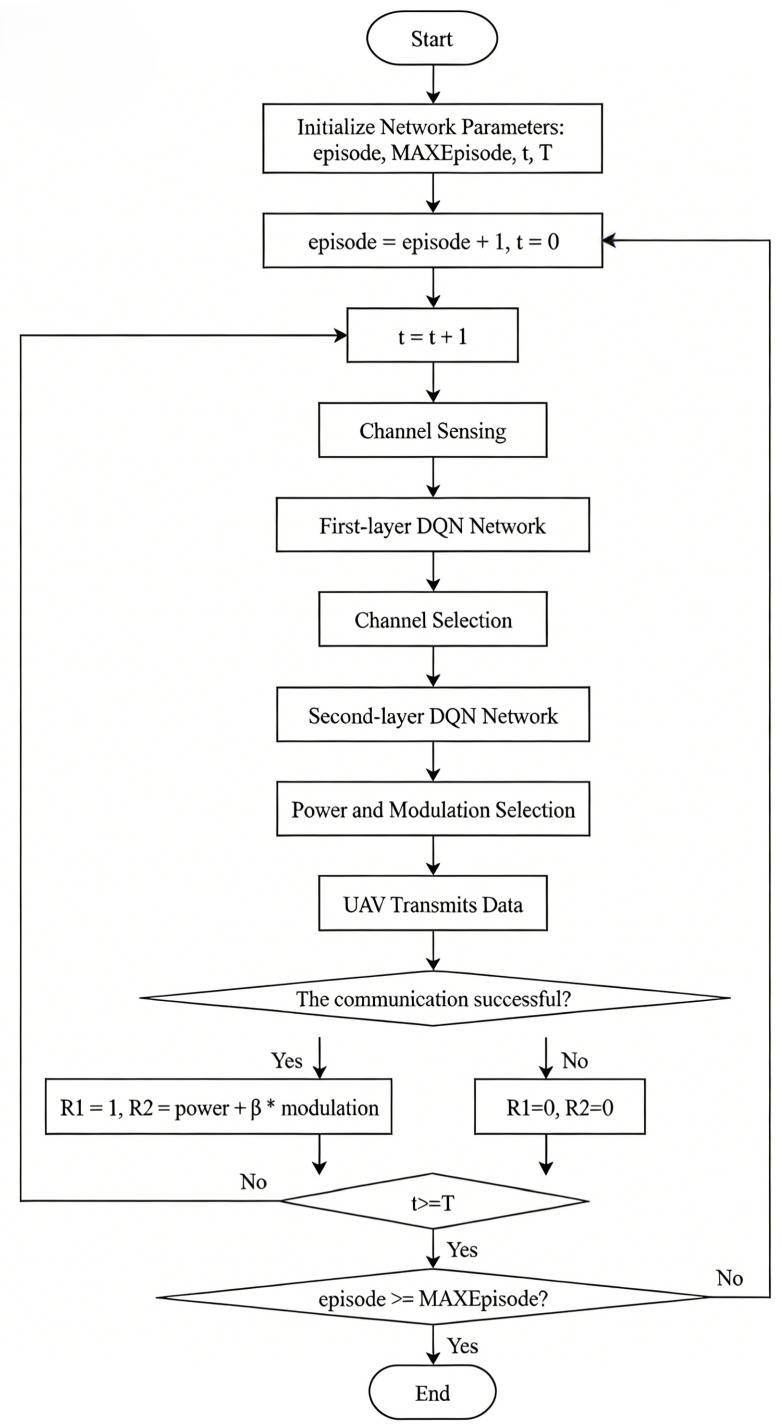
Process Framework of the Anti-Jamming Method Based on Hierarchical Reinforcement Learning.

Figure 8 illustrates the overall workflow of the hierarchical reinforcement learning algorithm.

### 3.4. Federated Learning-Enabled Intelligent Anti-Jamming Decision Algorithm for UAV Swarms

The proposed algorithm integrates an AMSM into the federated learning framework. In this approach, UAVs share only model parameters rather than raw data, thereby ensuring data privacy and security. Meanwhile, hierarchical reinforcement learning is employed to address the curse of dimensionality that arises in multi-domain decision scenarios. The overall process of the federated learning-based intelligent anti-jamming decision-making method for UAV swarms is illustrated in Algorithm 1. Initially, the algorithm sets up relevant decision parameters, including the number of iterations, failure counts, and intra-cluster self-update rounds. After initializing the communication environment and spectrum states, the cluster head distributes the initial network model to UAVs within the cluster. Each UAV then updates its local model according to the current environmental conditions and sends the updated model parameters back to the cluster head. The cluster head collects and caches these model parameters. Once the update condition is met, the cluster head updates the global model based on the predefined algorithm and distributes the new global parameters to all UAV nodes, simultaneously guiding subsequent communication decisions. This iterative process continues until the maximum number of iterations is reached.

The procedure of the adaptive model synchronization mechanism is presented in Algorithm 2. First, the cluster head initializes a buffer for storing UAV model parameters and an array for recording the actual number of received models in each round. It also sets an initial expected number of received models. Subsequently, the cluster head continuously receives model parameters from UAVs until the predefined update time is reached. At this point, the cluster head records the number of models stored in the buffer and adds this value to the collection of received model counts. Based on the relationship between the current buffer size and the expected number of received models, the cluster head adaptively adjusts the expected value for the next iteration.
**Algorithm 1** DQN-Based Multi-Domain Federated Intelligent Anti-Jamming Decision-Making Algorithm**Input:** Training data, experience replay buffer**Output:** Optimal policy estimation π1:Initialize the communication environment2:Initialize the DQN state space and action space3:Initialize the agent parameters based on the experience replay buffer size, discount factor, exploration rate, and learning rate4:The cluster head distributes the initial network model to all UAVs within the cluster5:Initialize the model buffer Buffer6:**for** cluster-head waiting time *t* from 0 to T−1 **do**7:    Each UAV updates its local model based on the hierarchical DQN framework described in Section 3.3.8:    **if** the UAV reaches the maximum number of local updates **then**9:        The UAV transmits the updated model to the cluster head10:       **if** the Buffer is not full **then**11:           The cluster head stores the received model into Buffer12:        **end if**13:    **end if**14:**end for**15:**for** each federated learning round *i* from 0 to N−1 **do**16:    Retrieve the expected number of models from buffer for global model updating17:    All UAV nodes terminate local training18:    The cluster head distributes the updated global model to all UAV nodes19:    After UAVs synchronously update their local networks, the cluster head broadcasts the decision information20:    **for** trans_count **do**21:        Intra-cluster transmitter transmits data22:        Receiver node receives data23:        **if** data transmission is successful **then**24:           **continue**25:        **else**26:           error_count←error_count+127:        **end if**28:        **if** error_count>error_count **then**29:           **break**30:        **end if**31:    **end for**32:**end for**33:**return** π

**Algorithm 2** Adaptive Model Synchronization Mechanism
**Input:** Model buffer Buffer, model count set Count**Output:** Expected number of received models num
1:Initialize Buffer, Count, and num2:**while** Buffer is not full or cluster head update time not reached **do**3:    UAVs transmit their locally trained model parameters to the cluster head4:    Cluster head stores the received models in Buffer5:
**end while**
6:Add the number of models in Buffer to Count7:**if** Count == num **then**8:    Maintain num unchanged9:
**else**
10:   num←Mean(Count)11:
**end if**
12:**return** num


## 4. Experiments and Result

### 4.1. Experimental Environment

The Experimental environment is established using the PyTorch 1.12.0 deep learning framework and the MATLAB R2021a simulation platform. The parameter settings of the single-UAV anti-jamming model are listed in Table 1. The communication system adopts an OFDM-based structure, and the simulation parameters follow the IEEE 802.11a WLAN PHY-layer standard [29], as shown in Table 2. The modulation schemes and their corresponding data transmission rates are provided in Table 3. The parameter configuration of the federated learning-based multi-UAV anti-jamming model is presented in Table 4. Four types of jamming patterns are considered in the simulation:

- Single-tone jamming: Channel 1 is continuously jammed.
- Multi-tone jamming: Channels 1, 3, 5, 7, and 9 are continuously jammed.
- Tracking jamming: If the UAV selects the same channel as in the previous time slot, it will certainly experience jamming. If the selected channel is adjacent to the previous one, there is a 0.5 probability of being jammed.
- Sweeping jamming: Starting from Channel 1, the jammer sequentially switches the interfered channel in each time slot, forming a periodic sweeping interference pattern.

In the DQN-based learning framework, the ε-greedy exploration parameter is set to ε=0.9 to ensure sufficient exploration in the dynamic and partially unknown jamming environment, while the learning rate is set to α=0.01 to balance convergence speed and training stability. These values are commonly adopted in DQN-based algorithms and were found to provide stable learning performance in our experiments.

### 4.2. Experimental Results and Analysis

To verify the effectiveness of the proposed federated learning-based multi-domain UAV swarm intelligent anti-jamming decision-making method, this study conducts a series of comparative simulations. Specifically, four representative algorithms are selected for benchmarking:

- the TGDJ-AJ algorithm—a multi-domain joint cognitive anti-jamming decision-making approach based on deep double Q-learning networks proposed in [30];
- the JAJA-PDQN algorithm—a novel anti-jamming method based on PER-DQN, proposed in [31];
- the conventional Q-learning algorithm;
- the random selection strategy.

To clearly evaluate the effectiveness of different components in the proposed framework, a two-stage comparative study is conducted. First, four representative anti-jamming methods are compared with the proposed multi-domain joint intelligent anti-jamming strategy without federated learning (MDJ-HDQN). This comparison aims to validate the effectiveness of the hierarchical multi-domain decision-making architecture. The evaluation metrics include convergence performance, transmission success rate, average data rate, and average power level.

Second, to further assess the performance gains introduced by federated learning, the proposed FL-HDQN is compared with the MDJ-HDQN model. Since FL-HDQN extends MDJ-HDQN by incorporating federated learning and adaptive model synchronization, this comparison isolates the impact of distributed collaborative learning on system performance under heterogeneous jamming conditions. The simulation investigates:

1. the impact of different numbers of local training episodes per UAV;
2. the communication success rate under federated and non-federated training conditions;
3. the influence of the adaptive model synchronization mechanism on communication success rate;
4. the effect of varying the number of UAVs in the network.

Through these comparative experiments, the superior performance and robustness of the proposed algorithm are effectively demonstrated.

#### 4.2.1. Comparative Analysis: MDJ-HDQN vs. Existing Methods

**Figure 9:**
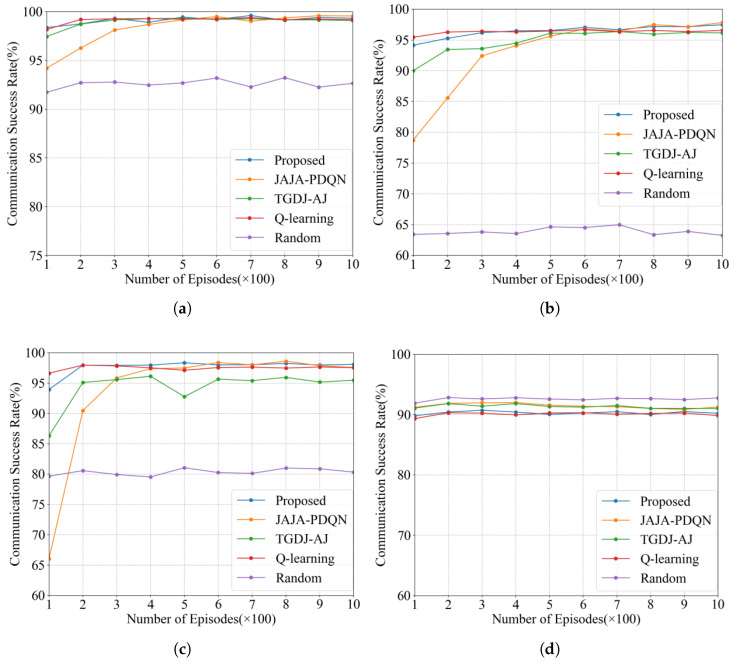
The communication success rate under different jamming conditions. (**a**) Communication success rate under single-tone jamming. (**b**) Communication success rate under multi-tone jamming. (**c**) Communication success rate under tracking jamming. (**d**) Communication success rate under sweeping jamming.

The communication success rates under the four types of jamming scenarios are illustrated in Figure 9. The proposed MDJ-HDQN algorithm demonstrates a significantly faster convergence speed in terms of communication success rate compared with JAJA-PDQN and TGDJ-AJ.

Under single-tone and multi-tone jamming, the convergence levels of the communication success rates are relatively close among all learning-based algorithms, except for the random selection method, which performs notably worse. In the case of sweeping jamming, the random strategy achieves a comparatively better result, as it can more effectively avoid this type of periodically varying wideband interference.

Table 5 summarizes the communication success rates of different anti-jamming algorithms after 1000 training episodes. Except for the sweeping jamming scenario, the proposed MDJ-HDQN consistently achieves a higher communication success rate than the other benchmark algorithms, demonstrating its strong anti-jamming capability and stable learning efficiency across various interference environments.

**Figure 10:**
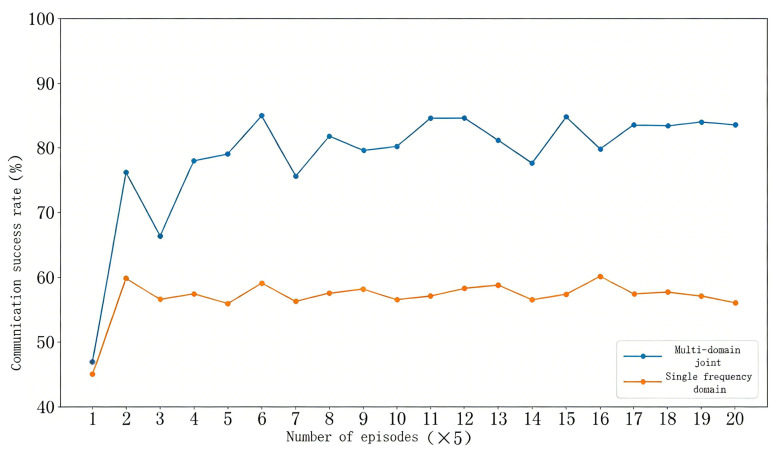
Communication success rate under full-band jamming.

Furthermore, when facing full-band jamming, where every channel experiences mild interference, adopting a fixed transmission power and modulation scheme results in the performance shown in Figure 10. The results indicate that the UAV communication success rate fluctuates around 55% under such static configurations. In contrast, the method proposed in this study dynamically adjusts transmission power and modulation schemes to cope with complex communication environments. Even under the extreme condition of full-band jamming, the proposed approach effectively mitigates interference and significantly improves the communication success rate to approximately 80%. This experimental result highlights the crucial role of adaptive communication parameter adjustment in maintaining efficient and stable UAV communications.

#### 4.2.2. Comparative Analysis: FL-HDQN vs. MDJ-HDQN

(1) Comparison of UAV local training episodes

**Figure 11:**
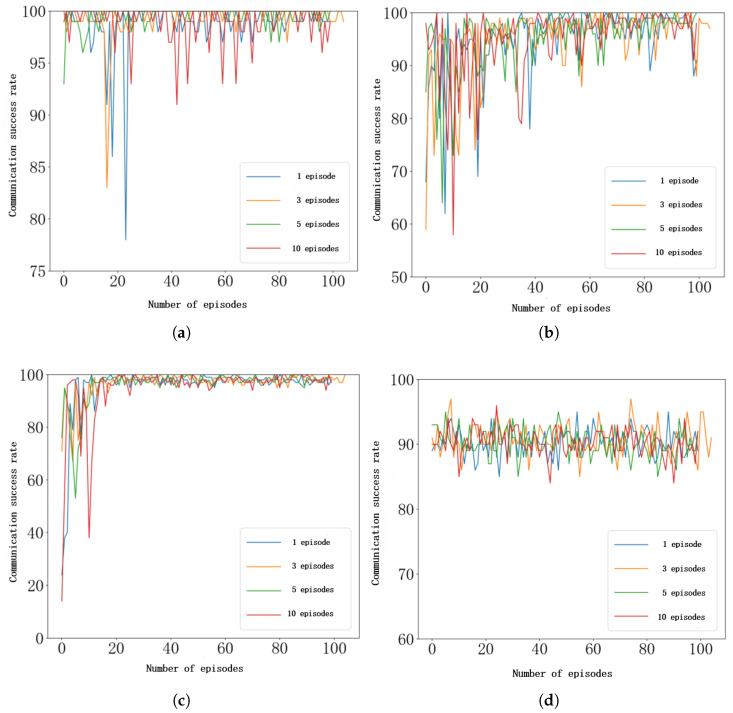
Effect of the number of UAV local training episodes under federated learning. (**a**) Communication success rate under single-tone jamming. (**b**) Communication success rate under multi-tone jamming. (**c**) Communication success rate under tracking jamming. (**d**) Communication success rate under sweeping jamming.

During the federated learning process, each UAV performs local self-training, and the number of local training episodes per UAV can significantly influence the overall training outcome. In this study, the number of local training episodes is set to 1, 3, 5, and 10, and the results are illustrated in Figure 11. As shown, when UAVs perform only 1 episode of local training before model aggregation, the performance is suboptimal. This is primarily because, within such a short training period, UAVs are unable to sufficiently explore and learn the complex characteristics of their operating environments, resulting in a globally aggregated model that lacks a comprehensive understanding of environmental dynamics. Conversely, when UAVs conduct 10 episodes of local training prior to aggregation, the performance also declines. Although each UAV achieves a more accurate fit to its local environmental features, excessive independent training causes the model parameters to become overfitted to localized conditions. Consequently, after aggregation, the global model exhibits reduced adaptability and generalization capability when exposed to diverse environmental conditions. The results corresponding to 3 and 5 episodes strike a more effective balance between local learning and global generalization. Considering the trade-off between model performance, generalization ability, and communication overhead, this study adopts 5 local training episodes as the default setting for subsequent experiments.

(2) Frequency channel selection for UAVs

To verify whether UAVs within a cluster make consistent channel selections under the same spectrum state after node model synchronization, this study simulates the channel selection results of cluster UAVs with and without federated learning.

Without federated learning, the percentage of n UAVs selecting the same channel is shown in Table 6. As observed from Table 6, in the absence of federated learning, the probability that cluster UAVs select the same channel is very low; the likelihood of 5 to 8 UAVs choosing the same channel is almost zero. In contrast, when federated learning is applied, the probability that all UAVs select the same channel reaches 1.

(3) Communication success rate with and without federated learning

**Figure 12:**
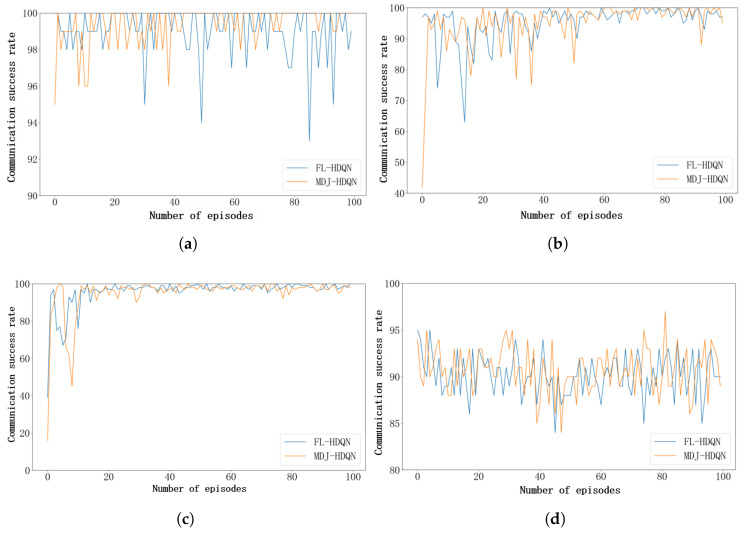
Comparative analysis of communication success rates of FL-HDQN and MDJ-HDQN under different jamming environments. (**a**) Communication success rate under single-tone jamming. (**b**) Communication success rate under multi-tone jamming. (**c**) Communication success rate under tracking jamming. (**d**) Communication success rate under sweeping jamming.

To further investigate the effectiveness of FL in UAV communication systems—particularly its capability in model aggregation and convergence—this study conducts a comparative analysis between a UAV swarm employing a federated learning–based anti-jamming strategy and a single UAV utilizing the MDJ-HDQN approach under four distinct jamming scenarios. In the FL experiments, the communication performance of a randomly selected UAV pair is evaluated, whereas for the MDJ-HDQN approach, the results are obtained from a single UAV pair performing independent training. The experiments encompass representative jamming types, including single-tone, multi-tone, sweeping, and tracking jamming, aiming to comprehensively assess the practicality and effectiveness of FL in real-world UAV communication interference environments. As illustrated in Figure 12a, under the relatively simple single-tone jamming scenario, the FL-based method maintains a high communication success rate, exhibiting no significant difference compared with the single-UAV MDJ-HDQN approach.

As illustrated in Figure 12b, the comparison of communication success rates between FL and the single-UAV MDJ-HDQN approach under multi-tone jamming reveals notable differences in their training dynamics. During the initial training phase, the FL-based model exhibits considerable fluctuations in communication success rate. This phenomenon arises because, in a distributed FL system composed of multiple UAVs, each UAV operates in distinct environments and performs various tasks, resulting in differences in the locally collected datasets. Although each UAV can train a local model tailored to its specific operational context, when these locally trained models are aggregated by the cluster head to form a global model, the substantial statistical discrepancies among the local datasets—known as the non-independent and identically distributed (non-IID) problem—can hinder the global model’s ability to generalize effectively across all UAV tasks. Consequently, the overall accuracy tends to decline during the early training stage. It is worth noting that although the non-IID data distribution among UAVs introduces performance fluctuations in the early training stage, the proposed federated learning framework with adaptive synchronization gradually alleviates this issue. Specifically, periodic global aggregation enables the shared model to capture common interference patterns across heterogeneous environments, while local updates preserve UAV-specific characteristics. As training proceeds, the adaptive synchronization mechanism balances local specialization and global generalization, thereby mitigating the adverse impact of non-IID data and leading to more stable communication performance under heterogeneous jamming conditions.

Figure 12c illustrates the comparison of communication success rates between the FL approach and the single-UAV MDJ-HDQN method under tracking jamming conditions. Similarly, during the early stages of training, the FL model exhibits greater fluctuations and a slightly slower convergence rate. However, once convergence is achieved, the communication success rate becomes more stable, indicating improved robustness and consistency in dynamic jamming environments.

Figure 12d presents the comparison of communication success rates between the FL approach and the single-UAV MDJ-HDQN method under sweeping jamming conditions. As shown, there is no significant difference between the two methods in this scenario.

**Figure 13:**
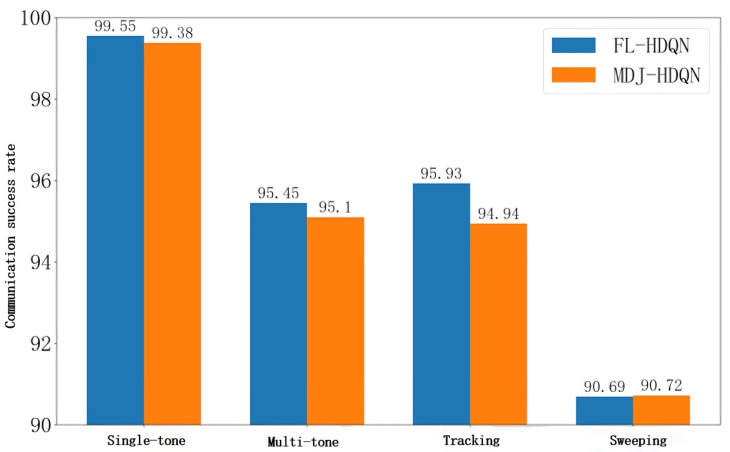
Average communication success rate of federated learning versus single-UAV under different jamming conditions.

Figure 13 illustrates the average communication success rates of FL-HDQN and MDJ-HDQN after 100 training episodes under four different jamming scenarios. With the exception of sweeping jamming, where the average success rate shows little change, the communication success rates under the other three types of jamming exhibit noticeable improvement after applying federated learning. These results indicate that, apart from sweeping jamming, the federated learning-based approach effectively enhances the communication efficiency and success rate of UAVs when encountering various types of jamming, demonstrating its strong adaptability and collaborative learning benefits in dynamic interference environments.

(4) Communication success rate with and without the adaptive model synchronization mechanism

In a real-world UAV swarm environment, the physical separation between UAVs leads to non-uniform interference exposure, meaning that each UAV may experience different interference conditions. In this experiment, all UAVs are subjected to multi-tone jamming; however, half of the UAVs experience continuous jamming on channels 1, 3, 5, 7, and 9, while the other half encounter continuous jamming on channels 2, 4, 6, 7, and 9. This configuration represents a hybrid jamming scenario, where multiple distinct interference patterns coexist within the UAV network.

**Figure 14:**
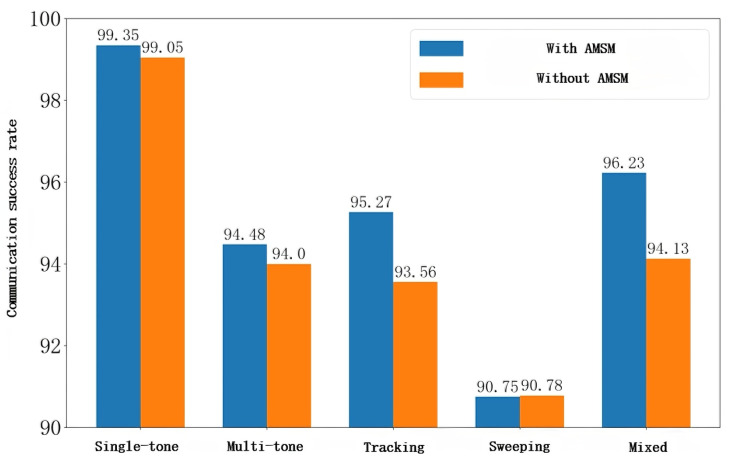
Communication success rate with and without the synchronization mechanism under five jamming conditions.

Figure 14 compares the average communication success rates with and without the adaptive model synchronization mechanism after 100 training episodes under four different jamming scenarios. Similar to previous results, the average success rate under sweeping jamming remains largely unchanged, while in the other three types of jamming, the inclusion of the AMSM leads to a notable improvement in communication success rates.

This demonstrates the effectiveness of the federated learning approach in handling most types of jamming, significantly enhancing UAV communication performance and the probability of successful mission completion. In particular, the improvements observed under tracking jamming and hybrid jamming are more pronounced, highlighting the strong capability of federated learning to cope with complex and dynamically varying interference environments.

(5) Analysis of results with varying UAV swarm sizes

**Figure 15:**
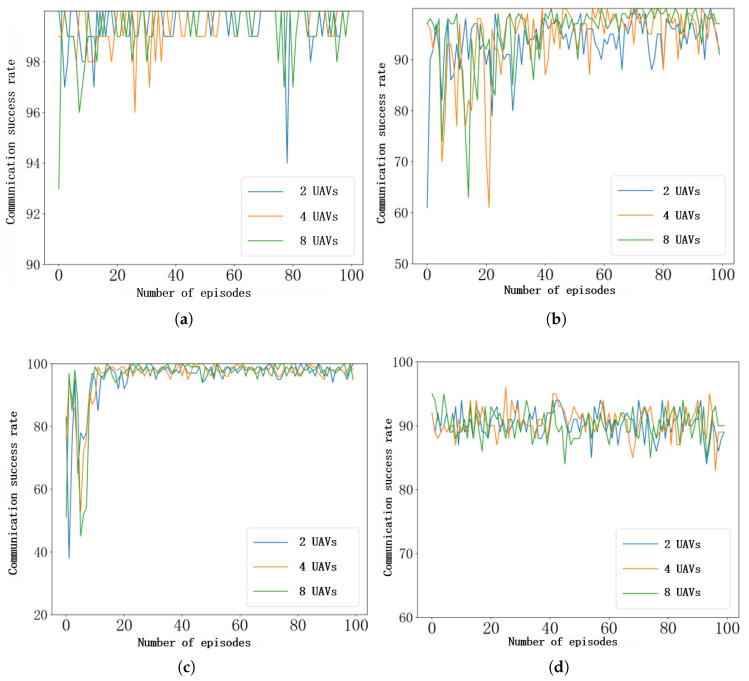
Effect of varying UAV swarm sizes on FL-HDQN. (**a**) Communication success rate under single-tone jamming. (**b**) Communication success rate under multi-tone jamming. (**c**) Communication success rate under tracking jamming. (**d**) Communication success rate under sweeping jamming.

**Figure 16:**
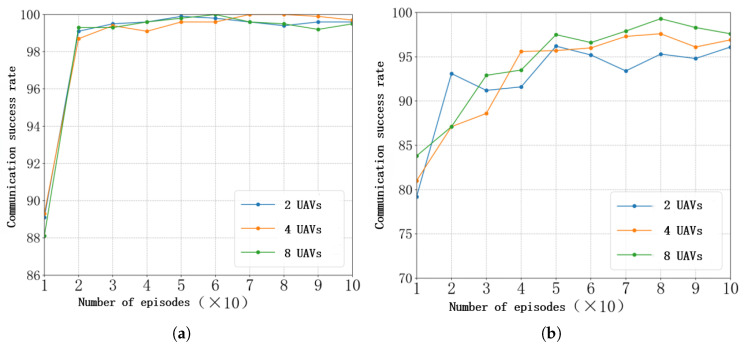

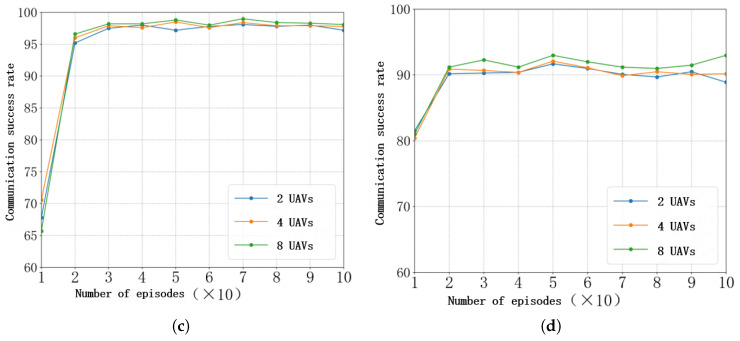
Average communication success rate of FL-DQN with different UAV swarm sizes. (**a**) Communication success rate under single-tone jamming. (**b**) Communication success rate under multi-tone jamming. (**c**) Communication success rate under tracking jamming. (**d**) Communication success rate under sweeping jamming.

Figure 15 and Figure 16 illustrate the impact of varying UAV swarm sizes on the performance of federated learning. Experimental results indicate that, during the early stages of training, a larger number of participating UAVs leads to greater model fluctuation. This phenomenon occurs because, in the initial learning phase, a larger swarm introduces more diverse local information, and when these heterogeneous updates are aggregated, they cause temporary instability in the global model. However, as training progresses and the model converges, a greater number of UAVs contribute to a more comprehensive understanding of the overall environment, resulting in reduced post-convergence oscillations and a significant improvement in communication success rate. This demonstrates that while larger UAV swarms may introduce early-stage training volatility, they ultimately enhance the robustness and generalization ability of the federated learning framework.

## 5. Conclusions

This study focuses on anti-jamming decision-making in multi-domain cooperative UAV swarms and develops an effective interference mitigation strategy. First, an adaptive model synchronization mechanism is integrated into the federated learning framework, enabling UAVs to share model parameters instead of raw data. This approach not only preserves privacy during communication but also ensures synchronized and coordinated decision-making across the swarm operating on shared frequency channels. As a result, communication security, coherence, and training convergence speed are significantly improved. Furthermore, this work introduces a multi-domain joint anti-jamming method based on a hierarchical DQN. By decomposing the high-dimensional action space into multiple hierarchical sub-decision problems across the frequency, power, and code domains, the proposed approach effectively reduces the search complexity faced by a single-layer network in large-scale state-action spaces. At the same time, it allows UAVs to adaptively adjust their operational parameters, ensuring that high communication success rates are maintained even under severe electromagnetic interference conditions. While federated learning avoids sharing raw data, the exchange of model parameters may still introduce potential information leakage risks. In anti-jamming communication scenarios, leaked parameters could be exploited to infer or counteract the learned strategies. Therefore, future work will focus on incorporating encryption and secure aggregation mechanisms into the proposed FL-HDQN framework, in order to enhance parameter-level privacy protection and improve robustness against adversarial inference attacks.

## Figures and Tables

**Table 1:** Single-UAV Anti-Jamming Model Parameters.

Parameter Name	Parameter Value
Epsilon (ε)	0.9
Lambda (λ)	0.9
Alpha (α)	0.01
Memory Capacity	10,000
Number of Time Slots per Episode	100
Number of Channels (*c*)	10
Power Levels (*p*)	4
Modulation Levels (*m*)	4
Weight α	0.15
Weight β	0.1

**Table 2:** OFDM Simulation Parameters.

Parameter Name	Parameter Value
Modulation Scheme	BPSK, QPSK, 16QAM, 64QAM
Number of Subcarriers	52
Number of Pilots	4
OFDM Symbol Duration (μs)	4
Guard Interval Duration (ns)	800
Subcarrier Spacing (kHz)	312.5
Signal Bandwidth (MHz)	16.66
Channel Spacing (MHz)	20

**Table 3:** Modulation Scheme Simulation Parameters.

Modulation Scheme	Data Rate (Mbps)
BPSK	6
QPSK	12
16QAM	24
64QAM	48

**Table 4:** Federated Learning-Based Multi-UAV Anti-Jamming Model Parameters.

Parameter Name	Parameter Value
Number of Intra-Cluster UAVs (excluding cluster head)	8
Number of Cluster Heads	1
Total Number of Communications for Task Completion	1000
Error Count	3
Number of Time Slots per Episode	100
Number of Channels (*c*)	10
Power Levels (*p*)	4
Modulation Levels (*m*)	4
Weight α	0.15
Weight β	0.1

**Table 5:** Communication success rate of different anti-jamming methods after 1000 episodes (%).

Anti-Jamming Method	Single-Tone Jamming	Multi-Tone Jamming	Tracking Jamming	Sweeping Jamming
Proposed	99.26	96.50	97.76	90.40
JAJA-PDQN [23]	98.50	93.31	93.86	91.57
TGDJ-AJ [21]	99.09	94.95	94.45	91.43
Q-learning	99.25	96.43	97.55	90.19
Random	92.69	63.95	80.41	92.69

**Table 6:** Probability of UAVs selecting the same frequency channel without FL.

Number of UAVs	Single-Tone Jamming	Multi-Tone Jamming	Tracking Jamming	Sweeping Jamming
2	0.29	0.2	0.24	0.22
3	0.12	0.15	0.11	0.08
4	0.02	0.05	0.04	0.03
5	0	0	0.02	0.01
6	0	0	0	0
7	0	0	0	0
8	0	0	0	0

## Data Availability

The original contributions presented in this study are included in the article. Further inquiries can be directed to the corresponding author.
